# Defining a theoretical framework for a quality referral at the primary–secondary care interface: a systematic scoping review with qualitative content analysis

**DOI:** 10.3399/BJGP.2025.0304

**Published:** 2026-05-19

**Authors:** Samatar Osman, Rosie Harrison, Claire Burton, Samantha L Hider, Henna Raiyat, Victoria K Welsh, Alice Faux-Nightingale

**Affiliations:** 1 School of Medicine, Keele University, Newcastle-under-Lyme, UK; 2 University Hospitals of North Midlands NHS Trust, Stoke-on-Trent, UK; 3 Haywood Academic Rheumatology Centre, Midlands Partnership University NHS Foundation Trust, Stoke-on-Trent, UK; 4 Department of Primary Care and Public Health, Brighton and Sussex Medical School, Brighton, UK

**Keywords:** primary health care, referral and consultation, secondary health care, general practitioners, specialist care

## Abstract

**Background:**

Referral from primary to secondary care is a crucial component of a GP’s role, yet the quality of referrals varies widely. Inadequate referral information can delay patient care, compromise outcomes, and strain healthcare resources. There is currently no universally accepted framework for what constitutes a quality referral.

**Aim:**

To define a theoretical framework for high-quality referrals between primary and secondary care, and to identify key components of effective referral practice.

**Design and setting:**

A systematic scoping review using a systematic search of Embase, CINAHL, and Medline databases was conducted to identify research published between July 1999 and August 2024. A qualitative content analysis describing the quality of GP referrals was conducted following Preferred Reporting Items for Systematic reviews and Meta-Analyses extension for Scoping Review guidelines.

**Method:**

Eligible studies included English-language publications with referral quality as a primary outcome, covering referrals from primary to secondary care. Studies were analysed inductively using qualitative content analysis to identify key attributes of effective referrals, which were grouped into overarching themes.

**Results:**

The search yielded 3461 studies; 54 were included in analysis. Four themes were identified: patient clinical characteristics (complete medical history, relevant investigations, and physical examination); clinical reasoning (clear referral indication, structured template use, guideline adherence, and appropriate urgency classification); patient factors (patient understanding, patient preference, and flow of information); and referral barriers (time constraints, limited specialty expertise, and educational needs). A quality-improvement checklist based on these findings was developed.

**Conclusion:**

Referral letters must balance detail and usability, taking a pragmatic approach to ensure clinical reasoning, relevant history, and patient involvement are communicated clearly, while avoiding unnecessary administrative burden.

**How this fits in**

Referral is a fundamental component of a GP role and is closely linked to quality of care. Concerns persist in secondary care around quality of referrals from primary care, and existing referral guidance is not specific to primary care. This scoping review and qualitative content analysis has outlined four key themes and developed a new quality-improvement checklist to support optimal referrals from primary to secondary care. This can be used in multiple referral pathways across the primary–secondary care interface, including in processes that seek specialist advice and guidance.

## Introduction

Referral is a fundamental component of the GP role, directly shaping patient experience, influencing clinical outcomes, and having an impact on the efficiency and costs within the broader healthcare system. The absence of a standardised referral framework contributes to healthcare inefficiencies, including delayed access to care, suboptimal resource use, and potential harm to patients; particularly vulnerable groups such as older adults.^1^ The act of referral often involves the transfer of clinical responsibility, making it a nuanced area of decision making. Primary care clinicians are required to navigate dual responsibilities: serving as patient advocates while also acting as gatekeepers of NHS resources — a role that continues to generate debate.^2^ The Royal College of General Practitioners’ pre-COVID-19 guidance on quality referrals highlights that demographic shifts and resource pressures have increasingly complicated this gatekeeping function. As a result, the quality of referrals is tightly linked to both the GP–patient relationship and broader system performance.^3^

Referrals from primary to secondary, or other specialist, care are made for diverse reasons: diagnostic clarification, initiation of specialist interventions, access to investigations unavailable in primary care, addressing patient anxiety, or acquiring expert input.^4^ Existing referral guidance remains generalist, typically recommending the inclusion of 'relevant' or 'necessary' information.^3^ However, the development of more tailored primary care-specific frameworks could enhance the consistency and quality of referrals, improving the patient journey, and resource allocation.

In the UK, the General Medical Council’s Good Medical Practice underscores that clinicians must deliver care of a high standard and refer when it benefits the patient, ensuring pertinent information is shared and that the recipient is appropriately qualified to manage the patient safely.^5^ The King’s Fund GP inquiry outlines three pillars of a high-quality referral: necessity — referrals made appropriately and without undue delay; destination — patients are sent to the correct service the first time; and process — the logistics of the referral are handled competently.^1^ This includes clearly written referral letters, engaging the patient in decision making, shared clarity on referral goals, and completing preliminary investigations where appropriate.

Nevertheless, concerns persist among secondary care clinicians regarding the adequacy of referral communications. Poorly constructed referral letters can delay triage, investigations, and treatment, thus compromising care delivery.^6,7^ Common deficiencies include incomplete symptom descriptions, omitted treatment history, unclear urgency, and minimal justification for referral.^8,9^ Underlying contributors range from workflow interruptions and time pressures to the absence of structured templates and feedback loops.^3^

Referral management centres have been implemented across parts of the NHS to reduce variation and curb demand, yet their effectiveness remains contested. Evidence indicates that although they may filter some inappropriate referrals, they often add cost and administrative burden without consistently reducing outpatient activity.^10^ Furthermore, concerns persist that referral management centres risk undermining GP autonomy and reducing referrals to a numbers-driven exercise, rather than a learning opportunity focused on patient care.^11^ Peer-led review models with consultant input have demonstrated greater promise in enhancing referral quality and clinician engagement. Additionally, the changing composition of the primary care workforce, including the increasing role of advanced nurse practitioners and other allied health professionals, further shapes referral practices and expectations.^12^

This systematic scoping review, employing qualitative content analysis, aims to define a theoretical framework for the referral process at the primary–secondary care interface.

## Method

### Inclusion and exclusion criteria

**Figure 1.**
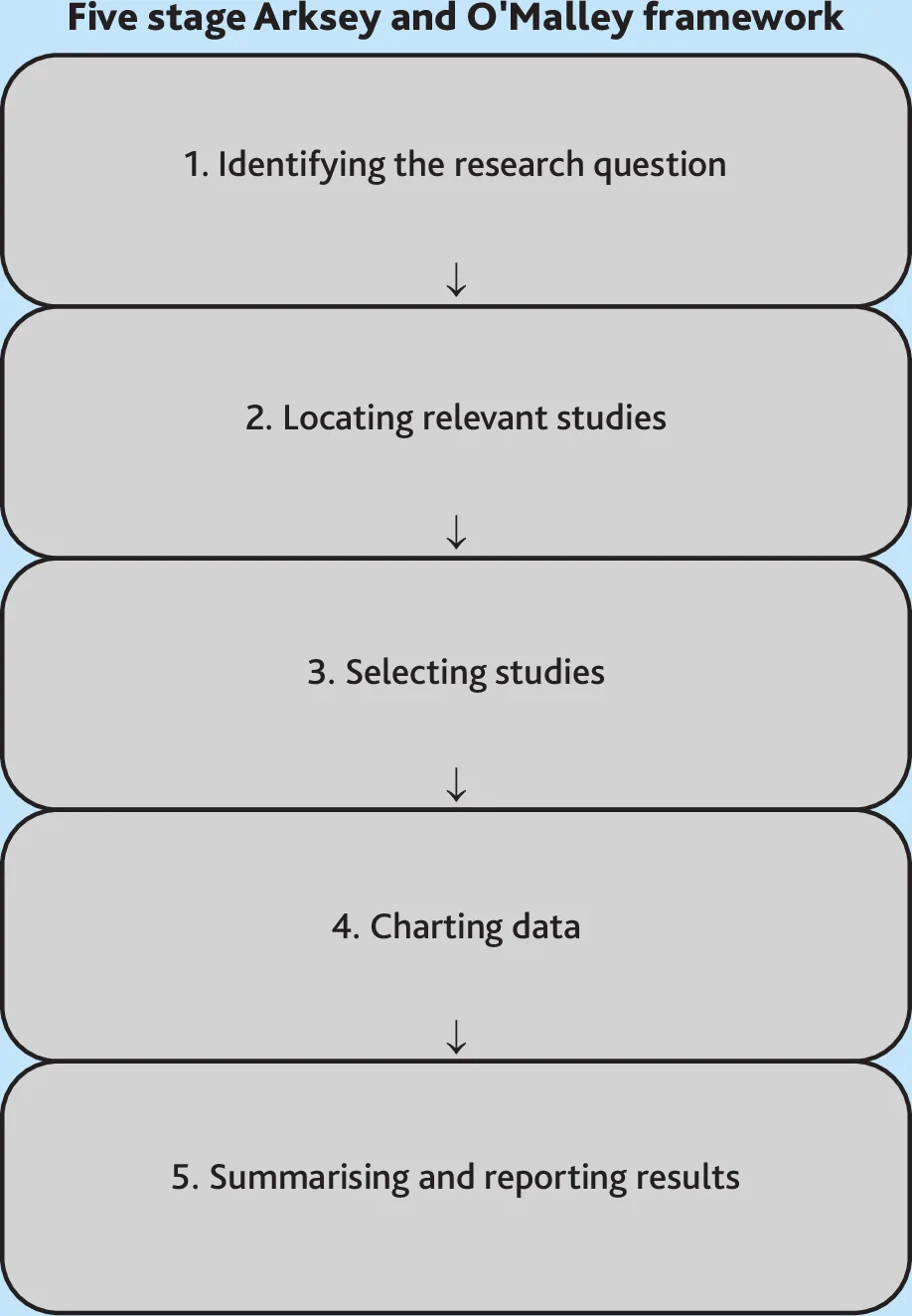
Diagram of the scoping review framework.^15^

A systematic scoping review was conducted in line with Preferred Reporting Items for Systematic reviews and Meta-Analyses extension for Scoping Reviews guidelines (see Supplementary Information S1).^13^ A systematic scoping review is a rigorous evidence synthesis method that maps the breadth and nature of research on a given topic, aiming to clarify key concepts, identify knowledge gaps, and summarise existing evidence without typically undertaking formal critical appraisal. It is particularly useful when the research question is broad or exploratory in nature.^14^ The five-stage Arksey and O’Malley framework was followed (Figure 1).^15^ The process began with identifying a broad research question to map the quality of referrals across the primary–secondary care interface. A systematic search strategy was developed to locate relevant studies, with eligibility criteria applied during study selection to ensure rigour and transparency. Data from included studies were charted using a structured extraction form to capture key variables such as referral content, processes, and outcomes. Finally, results were summarised using qualitative content analysis to identify overarching themes and gaps in the evidence.^15^

### Data sources and search strategy

A comprehensive search of Embase, CINAHL, and Medline databases was undertaken by two authors (first and fifth authors) covering studies from July 1999 to 21 August 2024. The outputs from key agencies were also considered in the grey literature search. The complete search terms are detailed in Box 1. The review protocol was preregistered on the Open Science Framework (https://osf.io/mwrzx) to ensure transparency and reduce duplication.^16^

**Box 1.** Search strategy

Category	Key words
**Population**	general W0 pract* OR GP* OR family W0 physician* OR “primary care” W0 physician* OR family W0 pract* OR family W0 doctor*
**Referral**	refer*
**Setting**	“primary care” OR primary W0 pract* OR “family medicine”

Included studies focused on referrals from primary care to secondary services (including specialist, imaging, or screening) via any method (electronic, paper, or verbal). Studies were excluded if not in English, lacked referral quality as a primary outcome, or did not involve both primary and secondary care. Disagreements on eligibility were resolved through discussion. Box 2 outlines full inclusion/exclusion criteria.

**Box 2.** Eligibility criteria

Inclusion	Exclusion
Time period July 1999 to August 2024	Any outside these dates
All referral methods (for example, paper, verbal, and electronic)	Not applicable
Studies available in English language	Non-English written studies
Referral quality as a primary outcome measure	Studies focusing on referral factors, for example, number of referrals, referral patterns, and perspectives
Referrals directly from primary to secondary care services	Studies that do not specify both primary and secondary care

### Study selection process

Six reviewers (first, second, third, fifth, sixth and senior authors) screened titles and abstracts, with duplicates removed. Full texts were assessed for eligibility, and discrepancies were resolved by discussion. The first author also screened reference lists for additional studies. Rayyan software supported this process.^17^

### Data extraction and results synthesis

Data were extracted into a Microsoft Excel sheet by the first and fifth author, with disagreements resolved collaboratively. Key variables included study design, location, demographics, referral content, process, outcomes, and identified quality indicators.

Qualitative content analysis followed Elo and Kyngäs’s inductive approach to identify themes related to the referral process and what constitutes a quality referral.^18^ Data were coded through a three-phase model: preparation, organisation, and reporting. Codes were refined iteratively by the team, enhancing reliability. Thematic summaries and a thematic analysis table were produced; a worked example of how data extracts contributed to the final themes is provided in Supplementary Box S1. Findings informed a referral-quality checklist, which is being piloted for use in local quality-improvement initiatives.

As a scoping review, a formal quality appraisal of included studies was not undertaken as it was not relevant for the aims and purpose of the review, which was to map the available evidence, not evaluate its rigour. This is consistent with accepted methodological frameworks for scoping reviews that aim to map the evidence rather than evaluate study rigour.

### Patient and public involvement

A patient and public involvement and engagement (PPIE) group collaborated with the research team to shape the review question and guide thematic analysis, ensuring the patient perspective was integrated. A summary of their input is found in the discussion.

## Results

### Study selection

**Figure 2.**
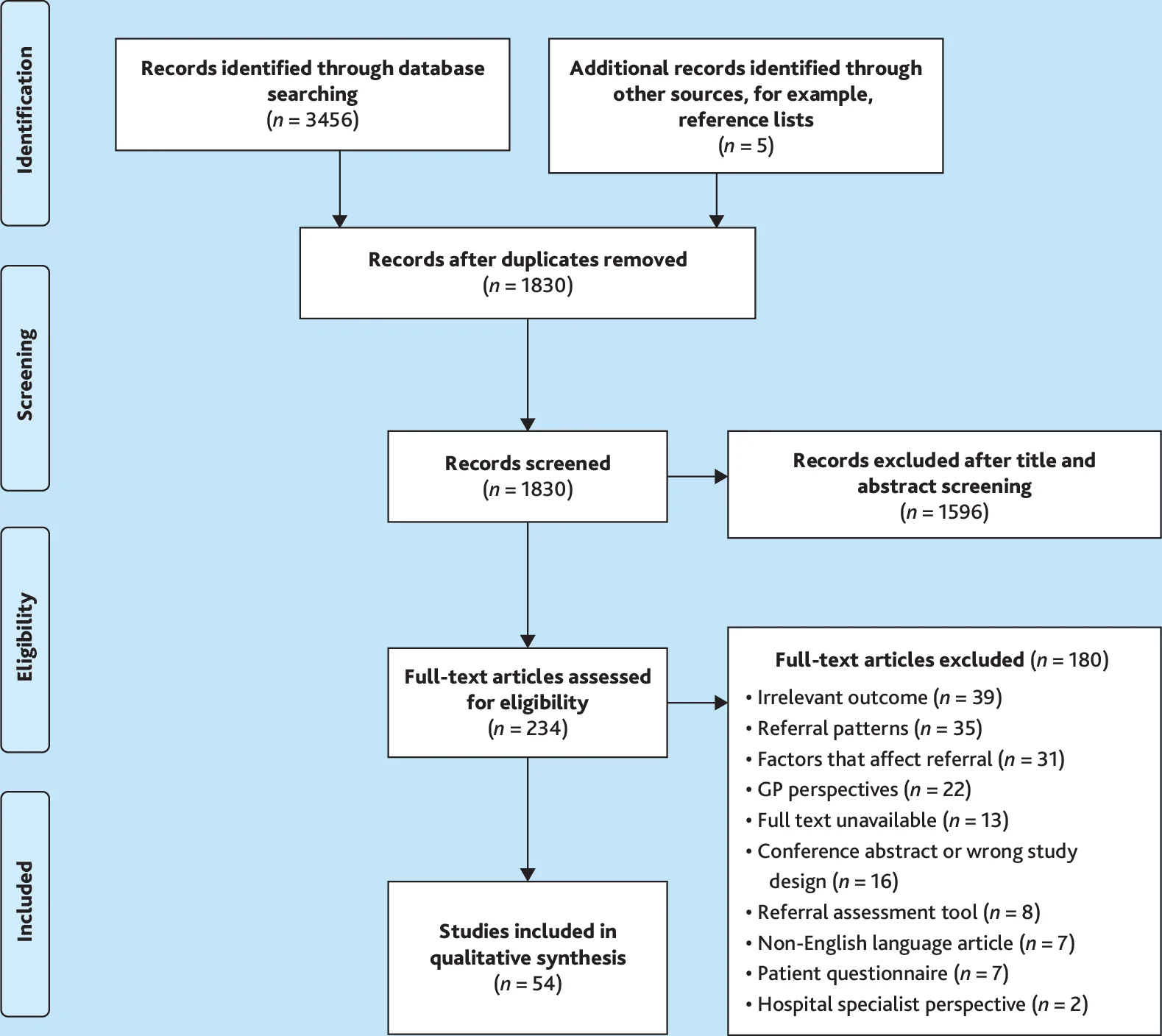
PRISMA flow diagram.

The initial search from three databases yielded a total of 3456 results, with a further five studies identified from reference checking. Following the removal of duplicates, 1830 studies were imported into Rayyan for title and abstract screening.^17^ There were 1596 studies that were excluded based on the inclusion criteria, resulting in 234 proceeding to full-text assessment. A further 180 studies were then excluded, resulting in 54 studies being included in this scoping review.^19–72^ The full PRISMA flowchart is provided in Figure 2.

### Study characteristics

A detailed sample description of the included articles is tabulated in Supplementary Table S1. In the 54 studies, the most frequent country of origin was the UK (*n* = 19, 35%), followed by Norway (*n* = 5, 9%), and Australia (*n* = 5, 9%). GP referrals to mental health services comprised the largest subset (*n* = 11, 20%), followed by accident and emergency (*n* = 7, 13%), ear, nose, and throat (ENT, *n* = 4, 7%), and imaging services (*n* = 4, 7%). The most frequent design was an audit or quality-improvement project (*n* = 17, 31%), followed by observational studies (*n* = 14, 26%) and cross-sectional studies (*n* = 11, 20%), and the other studies used a different design (*n* = 12, 22%). Table 1 summarises the key study characteristics.

**Table 1.** A summary of study characteristics (*N* = 54)

Characteristic	Study, *n*
**Geographical region**	
UK	19
Norway	5
Australia	5
New Zealand	4
Republic of Ireland	3
Canada	2
Saudi Arabia	2
Other	14
**Referral destination**	
Mental health services	11
Accident and emergency	7
More than one specialty	6
Ear, nose, and throat	4
Imaging services	4
Dermatology	3
Obstetrics and gynaecology	2
Genetic services	2
Respiratory	2
Memory clinic	2
Learning disability services	2
Other	9
**Study type^a^**	
Audit or quality-improvement study	17
Observational study	14
Cross-sectional study	11
Other or unspecific study design	12

### Content category and theme descriptions

Codes generated were used to create four overarching themes and twelve distinct sub-themes. Each theme is individually described, in no specific order, and collectively visually presented in Box 3.

**Box 3.** Overview of the four themes and their respective sub-themes

Theme and sub-themes	Description
**Patient clinical characteristics**	
Complete medical history	Clear use of full medical history including medical, family, drug, social, functional, travel, and allergy history. Including those relevant to referral destination
Relevant investigations	All relevant investigations (including those previously completed and trends) including, for example, bloods, imaging, and vital signs relevant to the referral specialty destination and differential diagnosis
Physical examination	Completion and documented physical examination relevant to the referral specialty (for example, ear, nose, and throat, ophthalmology, dermatology, and gynaecology)
**Clinical reasoning**	
Clear referral indication	To include referral reason or question to the secondary care professional (for example, diagnostic uncertainty, further investigations, optimisation of patient care, and consideration for surgery)
Structured template use	Use of standardised referral template, ideally electronic with features such as auto-fill of key information and ‘tick box’ features
Guideline adherence	Appropriateness of referral with reference to local and/or national guidelines for the referral destination
Appropriate urgency classification	Appropriate referral urgency, including use of the 2-week wait (cancer fast-track) or emergency referral pathway as needed. This needs to be stated on the referral form, and if multiple referral forms are available, to use the appropriate urgency indicator template
**Patient factors**	
Patient understanding	Patient awareness of the referral including clinical reason, expectations, and likely outcomes
Patient preference	Patient involvement in the referral process including documenting patient preferences in the referral
Flow of information	Factors including time to referral and ensuring clinical information, decisions, investigations, and relevant outcomes are relayed back to the GP. This ensures no repeat unnecessary referral or clinical errors are made
**Referral barriers**	
Time constraints	Pressures of general practice and if it is realistic for GPs to produce consistent, quality, detailed referrals in the limited admin time they have
Limited specialty expertise and educational needs	Importance of education and training in certain specialities

### Theme 1: patient clinical characteristics

Patient clinical characteristics were cited in 38 articles ^19,20,22–24,26,27,29–41,44,45,51,54–57,60–65,67–69,71,72^ as crucial to safe, effective referrals. Three sub-themes emerged: complete medical history, relevant investigations, and physical examination.

Eleven studies highlighted the frequent omission of comprehensive histories^19,22,40,44,45,55,56,60,61,65,67^ — including presenting complaint, medical and drug history, social history, and allergies. For instance, in ENT referrals for chronic unmanaged rhinosinusitis, only 22% included information on symptom duration.^44^

Referral letters often lacked physical examination findings. Da Casa *et al* identified joint examination documentation as a basic expectation in Spanish orthopaedic referrals.^47^ Similarly, Ong *et al* found <10% of Australian gastroenterology referrals included examination details, citing possible reasons such as lack of confidence or deference to the specialist.^45^

Pre-referral investigations were also commonly missing. Ong *et al* described this omission as problematic, leading to duplicated tests and patient harm (for example, radiation exposure).^45^

### Theme 2: clinical reasoning

The quality of clinical reasoning was a recurring focus in 47 articles, ^19–23,25–27,29–31,33–36,38–41,43–66,68,70–72^ with four sub-themes: clear referral indication, structured template use, guideline adherence, and appropriate urgency classification.

Referral questions were deemed central to quality for four articles.^22,45,52,60^ Odelola and Jabbar reported 95%–97% of GP mental health referrals in their UK study stated a reason, ranking this the most critical element.^52^

The importance of referral structure was cited by 45 articles ^19–23,25–27,29–31,33–36,38–41,43,44,46–59,61–66,68,70–72^ and structured templates, especially those integrated into electronic systems, improved referral consistency. Nash *et al* linked Australian improvements over 17 years to electronic medical records and standardised templates.^40^

Guideline-concordant referrals were associated with better appropriateness and outcomes in 17 articles.^21,25,30,31,33,36,43,44,46,47,56–59,61–63^ Kara *et al* found adherence improved intervention rates in their New Zealand study;^36^ by contrast, Krogh *et al* noted that 75.5% of Danish lumbar magnetic resonance imaging requests failed to meet appropriateness criteria.^30^

Urgency misclassification was reported in seven articles.^19,27,39,41,47,63,69^ Webb and Khanna in their UK study found inappropriate use of dermatology cancer fast-track referrals, potentially delaying care for patients with genuine malignancy.^63^

### Theme 3: patient factors

There were 17 studies ^20–22,27,31,32,39,43,47,51,56,57,62,65,67,68,71^ that stressed patient involvement and understanding as key elements of effective referrals. Sub-themes included patient understanding, patient preference, and flow of information.

Bodek *et al* reported that 41% of UK cardiology patients were unaware of being referred, and nearly one-quarter did not recall a GP visit, indicating persistent communication gaps.^68^

Documentation of patient preferences — particularly for vulnerable groups — was often inadequate. Vanpraag *et al* noted interpreter needs for refugee women in Australia were frequently omitted.^39^

Delays were also common. Tedajo *et al* highlighted UK menopause clinic referrals with waiting times of 2–16 months, eroding trust and engagement.^21^

### Theme 4: referral barriers

Nine articles considered referral barriers,^21,25–27,37,47,59,69,70^ which were complex and multifactorial, including time constraints, limited specialty expertise, and educational needs.

Raheja and Singh described UK GPs as overburdened and overwhelmed with administrative demands.^27^

Lack of familiarity with certain specialties and their referral guidelines, particularly highlighted by a dermatology department, led to increased inappropriate referrals. Hill *et al* advocated for targeted education in the UK to improve accuracy and care quality.^70^

Eight studies specifically recommended education for clinicians making referrals.^21,25,27,37,47,59,69,70^

### Quality-improvement checklist

**Figure 3.**
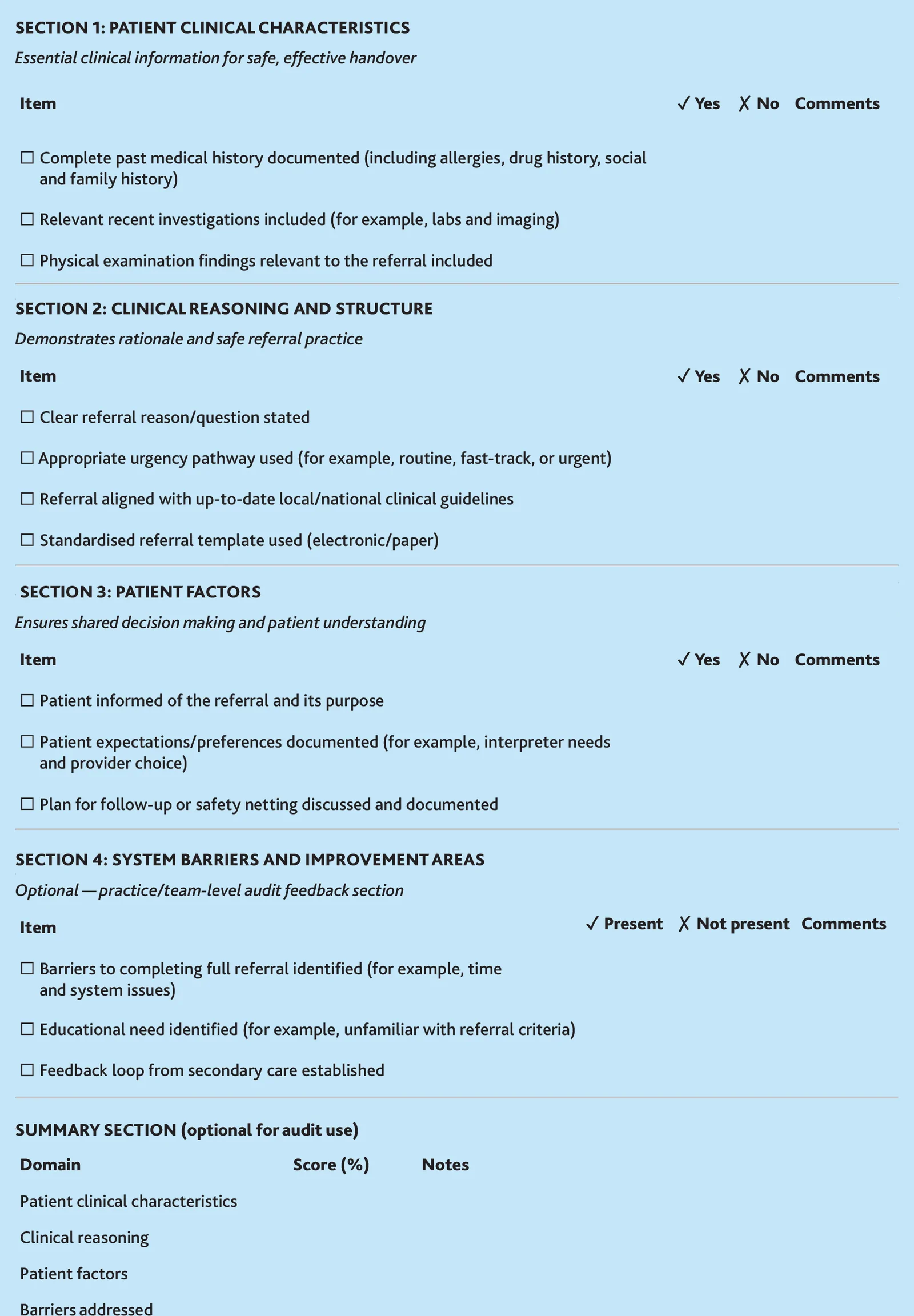
Quality-improvement checklist.

Drawing from these themes, the team developed a quality-improvement checklist for use in education and training, integration into clinical systems, and quality appraisal exercises (Figure 3). Initial evaluation of 20 referrals showed the tool supports systematic appraisal without highlighting any areas of referral that were not considered.

## Discussion

### Summary

From the 54 studies included in this review, 12 sub-themes and four overarching themes essential to understanding and optimising referrals from primary to secondary care were identified. These findings provide a theoretical framework defining the key components of a quality referral and highlight areas for improvement within the primary–secondary care interface.

### Strengths and limitations

A key strength of this review is its comprehensive synthesis across diverse healthcare systems, supported by patient involvement. However, it is limited by its exclusion of non-English studies. The inclusion of qualitative content analysis enabled the development of a theoretical framework that encapsulates diverse factors influencing referral quality. This structured approach provides a valuable foundation for policymakers, healthcare providers, and educators aiming to enhance referral pathways.^73^ Additionally, the review highlights specific elements that could be targeted for improvement, such as optimising referral templates, enhancing guideline adherence, and fostering better communication between primary and secondary care. Furthermore, its use with real-world data has been piloted by the research team.

Another key strength was the involvement of a PPIE group in shaping the research. Their input informed the formulation of the review question and provided valuable perspectives that helped guide the development and interpretation of the emerging themes. This collaboration ensured that patient voices were embedded throughout the review process, enhancing the relevance and applicability of the findings. Insights from the PPIE group offer further depth to these findings. The group raised concerns about the current lack of transparency in the referral process, with many participants reporting confusion and limited understanding of what happens once a referral is made. Several members expressed that they had never been actively involved in decisions about referrals or informed adequately about them, underscoring the importance of improved patient communication and education. The PPIE group identified specific populations — such as individuals with learning disabilities, autism, dementia, and those unfamiliar with the healthcare system — as particularly vulnerable to being overlooked or underserved in the current system.

Furthermore, most included studies originated from high-income countries, limiting generalisability to low- and middle-income healthcare settings where referral challenges may differ.^42^ Another limitation is the variability in study designs, which may introduce heterogeneity in findings. Finally, although the current study’s framework provides theoretical insights, further empirical validation through real-world implementation and testing in clinical settings is required.

### Comparison with existing literature

The four key themes identified — patient clinical characteristics, clinical reasoning, patient factors, and referral barriers — underscore the complexity of the referral process. Clinical information completeness is crucial, as missing patient history, investigations, or physical examination findings can hinder effective triage and contribute to unnecessary delays in patient care.^74,75^ Similarly, robust clinical reasoning, including clear referral reasoning and adherence to clinical guidelines, improves referral quality and enhances specialist decision making.^30,36^ A review by Winpenny *et al* that scoped interventions to improve care at the primary–secondary care interface highlights the benefits of clear communication between GPs and the specialist through access to telephone and emails, highlighting that good communication can improve care processes.^76^ As highlighted in a review by Saghdaoui *et al*,^77^ it is important to note that flexibility in referral processes is required to accommodate an individualised approach for patients with clinical complexity.

Patient factors also play a fundamental role in referral effectiveness. Ensuring patients understand the purpose of their referral and including patient preferences in decision making can enhance engagement and adherence to specialist recommendations.^78^ A meta-ethnographical synthesis by Sampson *et al* further highlights the importance of the consideration of patient perspective and experience.^79^ Moreover, referral barriers such as time constraints, lack of specialist knowledge among primary care clinicians, and feedback deficits from secondary care further complicate the process.^80–82^ Addressing these barriers is necessary for improving efficiency, minimising inappropriate referrals, and ensuring equitable access to specialist care.

Furthermore, PPIE group members highlighted the emotional and logistical burden of navigating the referral process, particularly when patients must complete multiple steps or face long waits before being seen. These insights aligned with the current study’s theme of ‘referral barriers’, drawing attention to the potential for disengagement and delays in care. The PPIE group acknowledged that while the proposed framework may introduce additional work for GPs in the short term, it could ultimately reduce inefficiencies — particularly if supported by robust IT systems that enable shared access to full patient records. Notably, the group advocated for involving patients more actively in the referral process itself, including the opportunity to contribute relevant information they believe should be passed to the specialist.

### Implications for research and practice

This scoping review provides a structured framework, derived inductively from international literature, for understanding and improving the referral process at the primary–secondary care interface. A quality referral comprises clear clinical reasoning, complete and relevant patient information, patient engagement, and minimised barriers that impede timely and appropriate specialist access. Addressing key deficiencies, including incomplete referral content, inadequate patient involvement, and structural inefficiencies, is essential to optimising referral pathways and enhancing patient outcomes. Future research should focus on implementing and evaluating interventions designed to refine bidirectional referral processes, particularly in diverse healthcare settings. Through targeted quality-improvement initiatives, healthcare systems can strive for more effective, patient-centred, and equitable referral practices.

## References

[1] Foot C, Naylor C, Imison C (2010). The quality of GP diagnosis and referral.

[2] Bi Y-N, Liu Y-A (2023). GPs in UK: from health gatekeepers in primary care to healthagents in primary health care. Risk Manag Healthc Policy.

[3] Marshall M, Gregory S, Bullard E (2018). Quality patient referrals: right service, right time.

[4] Ringberg U, Fleten N, Førde OH (2014). Examining the variation in GPs’ referral practice: a cross-sectional study of GPs’ reasons for referral. Br J Gen Pract.

[5] General Medical Council (2024). Delegation and referral. https://www.gmc-uk.org/professional-standards/the-professional-standards/delegation-and-referral.

[6] Piterman L, Koritsas S (2005). Part II. General practitioner-specialist referral process. Intern Med J.

[7] Street RL, Makoul G, Arora NK, Epstein RM (2009). How does communication heal? Pathways linking clinician-patient communication to health outcomes. Patient Educ Couns.

[8] Toleman J, Barras M (2007). General practitioner referral letters: are we getting the full picture?. Intern Med J.

[9] Blundell N, Clarke A, Mays N (2010). Interpretations of referral appropriateness by senior health managers in five PCT areas in England: a qualitative investigation. Qual Saf Health Care.

[10] Proctor N, Wake T, Roach C, Addala S (2017). Referral management: centres and services, and peer review. A rapid review of existing evidence. https://www.transformationpartners.nhs.uk/wp-content/uploads/2017/11/Rapid-review-Referral-managment-services.pdf.

[11] Gulland A (2017). All GP referrals should be subject to clinical peer review, says NHS England. BMJ.

[12] Groenewegen P, Heinemann S, Greß S, Schäfer W (2015). Primary care practice composition in 34 countries. Health Policy.

[13] Tricco AC, Lillie E, Zarin W (2018). PRISMA extension for scoping reviews (PRISMA-scr): checklist and explanation. Ann Intern Med.

[14] Munn Z, Peters MDJ, Stern C (2018). Systematic review or scoping review? Guidance for authors when choosing between a systematic or scoping review approach. BMC Med Res Methodol.

[15] Arksey H, O’Malley L (2005). Scoping studies: towards a methodological framework. Int J Soc Res Methodol.

[16] Foster ED, Deardorff A (2017). Open Science Framework (OSF). Jmla.

[17] Ouzzani M, Hammady H, Fedorowicz Z, Elmagarmid A (2016). Rayyan-a web and mobile app for systematic reviews. Syst Rev.

[18] Elo S, Kyngäs H (2008). The qualitative content analysis process. J Adv Nurs.

[19] Reiter-Campeau S, Moore F (2023). The role of the neurological examination in primary care referrals to neurology. Can J Neurol Sci.

[20] Hartveit M, Thorsen O, Biringer E (2013). Recommended content of referral letters from general practitioners to specialised mental health care: a qualitative multi-perspective study. BMC Health Serv Res.

[21] Tedajo Tsambou J, Bruce D, Holloway D, Rymer J (2024). A retrospective audit of general practitioner’s referrals to Guys and St Thomas’ specialist menopause clinic between 2021 and 2022. Post Reprod Health.

[22] Towobola A, Cunningham E, Cotter P (2015). Improving general practitioners’ referral details of antidepressant use for patients referred for depression to primary mental health care: re-audit. Ir J Psychol Med.

[23] Rogers C, Heatherington S, Carroll M (2013). An analysis of 100 referrals for depression from primary care to an adult mental health service. Ir J Psychol Med.

[24] Tanielian TL, Pincus HA, Dietrich AJ (2000). Referrals to psychiatrists. assessing the communication interface between psychiatry and primary care. Psychosom.

[25] Shaw I, Smith KMC, Middleton H, Woodward L (2005). A letter of consequence: referral letters from general practitioners to secondary mental health services. Qual Health Res.

[26] Taylor SC, Markar TN (2002). Audit of the quality of general practitioner referral letters to a learning disability service. Br J Dev Disabilities.

[27] Raheja S, Singh I (2001). Audit of referral letters from general practitioners to learning disabilities clinics. The British Journal of Development Disabilities.

[28] Aydin S, Crone MR, Siebelink BM (2023). Informative value of referral letters from general practice for child and adolescent mental healthcare. Eur Child Adolesc Psychiatry.

[29] Yuen J, Rashid M, Webber C, Prior M (2017). Is it possible to diagnose benign positional paroxysmal vertigo from primary care referrals to ENT clinic?. Hearing Balance Commun.

[30] Krogh SB, Jensen TS, Rolving N (2022). Appropriateness of referrals from primary care for lumbar MRI. Chiropr Man Therap.

[31] Peterson G, Frick J (2021). Extended roles in primary care when physiotherapist-initiated referral to x-ray can save time and reduce costs. Int J Qual Health Care.

[32] Harwood TJS, Paoloni RJ, Mitic SS (2015). Accuracy of medication lists on general practitioner referral letters to emergency departments for patients with congestive cardiac failure compared with other polypharmacy patients. J Pharm Pract Res.

[33] Logan GS, Dawe RE, Aubrey-Bassler K (2020). Are general practitioners referring patients with low back pain for CTs appropriately according to the guidelines: a retrospective review of 3609 medical records in newfoundland using routinely collected data. BMC Fam Pract.

[34] Allwood C, O’Brien A, Glue P (2019). Referrals from primary care to community mental health teams: what’s missing?. J Prim Health Care.

[35] Mash R, Steyn H, Bello M (2019). The quality of feedback from outpatient departments at referral hospitals to the primary care providers in the Western Cape: a descriptive survey. S Afr Fam Pract (2004).

[36] Kara S, Smart A, Officer T (2019). Guidelines, training and quality assurance: influence on general practitioner MRI referral quality. J Prim Health Care.

[37] Syed A, Large D (2003). Quality of gps’ referral letters to diabetes secondary care. Pract Diab Int.

[38] Yee A, Linton T, Lee M-S, Raimes S (2019). The factors that lead to a delay between general practitioner referral of symptomatic patients and specialist diagnosis of colorectal cancer: an audit in the bay of plenty district health board. N Z Med J.

[39] Vanpraag D, Dawson W, Bell B (2018). Enhancing general practice referrals for women of refugee background to maternity care. Aust J Prim Health.

[40] Nash E, Hespe C, Chalkley D (2016). A retrospective audit of referral letter quality from general practice to an inner-city emergency department. Emerg Med Australas.

[41] Hartveit M, Aslaksen A, Vanhaecht K (2015). Development and testing of an instrument in western Norway to measure the quality of referral information from primary care to specialised mental health care. Int J Care Coord.

[42] Corwin P, Bolter T (2014). The effects of audit and feedback and electronic referrals on the quality of primary care referral letters. J Prim Health Care.

[43] Dehghan A, Nowar MA, Molodynski A (2017). Improving the referral process from primary care to an AMHT. Prog Neurol Psychiatry.

[44] Su N, Cheang PP, Khalil H (2013). Do rhinology care pathways in primary care influence the quality of referrals to secondary care?. J Laryngol Otol.

[45] Ong SP, Lim L-T, Barnsley L, Read R (2006). General practitioners’ referral letters--do they meet the expectations of gastroenterologists and rheumatologists?. Aust Fam Physician.

[46] Anwar H, Harthi TA, Jaafar N (2024). Appropriateness of the emergency referrals made by primary care clinicians: a cross-sectional review of referral notes. Sultan Qaboos Univ Med J.

[47] da Casa C, Suárez ÁV, Asensio N, Blanco JF (2021). Quality assessment of orthopedic surgery referral request letters from primary care consultation: evaluation of a Spanish healthcare area. J Family Community Med.

[48] Marra E, van Rijsingen MCJ, Alkemade JAC (2021). The effect of a dermato-oncological training programme on the diagnostic skills and quality of referrals for suspicious skin lesions by general practitioners. Br J Dermatol.

[49] Faramarzi M, Shishegar M, Sabz GA (2019). Quality of referral letters written by family physicians to otologists - a peer assessment. Iran J Otorhinolaryngol.

[50] Ford BK, Kim D, Keay L, White AJ (2020). Glaucoma referrals from primary care and subsequent hospital management in an urban Australian hospital. Clin Exp Optom.

[51] Wojtowicz A, Larner AJ (2015). General practitioner assessment of cognition: use in primary care prior to memory clinic referral. Neurodegener Dis Manag.

[52] Odelola C, Jabbar F (2017). Re-audit of the contents of GP referral letters to general adult community psychiatrists. Psychiatr Danub.

[53] Eskeland SL, Brunborg C, Rueegg CS (2017). Assessment of the effect of an interactive dynamic referral interface (IDRI) on the quality of referral letters from general practitioners to gastroenterologists: a randomised cross-over vignette trial. BMJ Open.

[54] Janati A, Amini A, Adham D, Naseriasl M (2017). Assessing the quality of referral letters written by general practitioners: a cross-sectional study in rural Iran. Cad Saude Publica.

[55] Radford H, Fitzgerald P, Martin S, Johnson MI (2016). A service improvement project to review prescribing information provided by general practitioners for new referrals to a UK National Health Service hospital pain clinic: potential implications of CYP2D6 enzyme inhibition. Br J Pain.

[56] Scully P, O’Donnell B, Peters C (2016). Older patient hospital admissions following primary care referral: the truth is in the referring. Ir J Med Sci.

[57] Oosthuizen JC, McShane D, Kinsella J, Conlon B (2015). General practitioner ENT referral audit. Ir J Med Sci.

[58] Lucassen A, Watson E, Harcourt J (2001). Guidelines for referral to a regional genetics service: GPs respond by referring more appropriate cases. Fam Pract.

[59] Watson E, Austoker J, Lucassen A (2001). A study of GP referrals to a family cancer clinic for breast/ovarian cancer. Fam Pract.

[60] Abdelwahid HA, Al-Shahrani SI, Elsaba MS, Elmorshedi WS (2010). Patterns of referral in the family medicine department in Southeastern Saudi Arabia. Saudi Med J.

[61] Kozomara D, Galić G, Brekalo Z (2009). Abdominal pain patient referrals to emergency surgical service: appropriateness of diagnosis and attitudes of general practitioners. Coll Antropol.

[62] Culshaw D, Clafferty R, Brown K (2008). Let’s get physical! A study of general practitioner’s referral letters to general adult psychiatry--are physical examination and investigation results included?. Scott Med J.

[63] Webb JB, Khanna A (2006). Can we rely on a general practitioner’s referral letter to a skin lesion clinic to prioritise appointments and does it make a difference to the patient’s prognosis?. Ann R Coll Physicians Surg Can.

[64] Tuomisto L, Erhola M, Kaila M (2004). Asthma programme in finland: high consensus between general practitioners and pulmonologists on the contents of an asthma referral letter. Prim Care Respir J.

[65] McNeill A (2008). How accurate are primary care referral letters for presumed acute stroke?. Scott Med J.

[66] Al-Alfi M, Al-Saigul A, Abed-Elbast A (2007). Quality of primary care referral letters and feedback reports in Buraidah, Qassim region, Saudi Arabia. J Fam Community Med.

[67] Gran JT, Nordvåg BY (2000). Referrals from general practice to an outpatient rheumatology clinic: disease spectrum and analysis of referral letters. Clin Rheumatol.

[68] Bodek S, Ghori K, Edelstein M (2006). Contemporary referral of patients from community care to cardiology lack diagnostic and clinical detail. Int J Clin Pract.

[69] Grol R, Rooijackers-Lemmers N, van Kaathoven L (2003). Communication at the interface: do better referral letters produce better consultant replies?. Br J Gen Pract.

[70] Hill VA, Wong E, Hart CJ (2000). General practitioner referral guidelines for dermatology: do they improve the quality of referrals?. Clin Exp Dermatol.

[71] Kada S, Nygaard HA, Geitung JT (2007). Quality and appropriateness of referrals for dementia patients. Qual Prim Care.

[72] Tuomisto LE, Marina E, Minna K (2007). The Finnish national asthma programme: communication in asthma care – quality assessment of asthma referral letters. Evaluation Clinical Practice.

[73] AlHarthy SH, Al-MoundhrI M, Al-Mahmoodi W (2024). Referral process enhancement: innovative approaches and best practices. Asian Pac J Cancer Prev.

[74] Hendrickson CD, Saini S, Pothuloori A, Mecchella JN (2017). Assessing referrals and improving information availability for consultations in an academic endocrinology clinic. Endocr Pract.

[75] Smith PC, Araya-Guerra R, Bublitz C (2005). Missing clinical information during primary care visits. JAMA.

[76] Winpenny EM, Miani C, Pitchforth E (2017). Improving the effectiveness and efficiency of outpatient services: a scoping review of interventions at the primary-secondary care interface. J Health Serv Res Policy.

[77] Bolton Saghdaoui L, Lampridou S, Tavares S (2025). Interventions to improve referrals from primary care to outpatient specialist services for chronic conditions: a systematic review and framework synthesis update. Syst Rev.

[78] Dunlea R, Lenert L (2015). Understanding patients’ preferences for referrals to specialists for an asymptomatic condition. Med Decis Making.

[79] Sampson R, Cooper J, Barbour R (2015). Patients’ perspectives on the medical primary-secondary care interface: systematic review and synthesis of qualitative research. BMJ Open.

[80] Freedman S, Golberstein E, Huang T-Y (2021). Docs with their eyes on the clock? The effect of time pressures on primary care productivity. J Health Econ.

[81] Pathak KP, Montgomery A (2015). General practitioners’ knowledge, practices, and obstacles in the diagnosis and management of dementia. Aging Ment Health.

[82] Pannell LM, Tyrrell-Price J (2017). Communication between primary and secondary care. Br J Hosp Med (Lond).

